# Wheat Breeding, Transcription Factories, and Genetic Interactions: New Perspectives

**DOI:** 10.3389/fpls.2022.807884

**Published:** 2022-02-23

**Authors:** Richard B. Flavell

**Affiliations:** International Wheat Yield Partnership, College Station, TX, United States

**Keywords:** wheat, transcription factories, epigenetics, heterosis, breeding

## Abstract

Epistatic interactions and negative heterosis have been shown to be associated with interchromosomal interactions in wheat. Physical gene-gene interactions between co-regulated genes clustered in “transcription factories” have been documented, and a genome-wide atlas of functionally paired, interacting regulatory elements and genes of wheat recently produced. Integration of these new studies on gene and regulatory element interactions, co-regulation of gene expression in “transcription factories,” and epigenetics generates new perspectives for wheat breeding and trait enhancement.

## Introduction

Rates of wheat yield improvements have slowed in recent years to 0.5–0.9% per year, and there is much concern about achieving the additional yields necessary to feed the world using less land and with fewer inputs (Ray et al., 2013). There is, therefore, a need to continuously seek better and more informed ways of approaching plant breeding, including the making of commercial hybrids (Boeven et al., 2020; Gimenez et al., 2021). Wheat is a polyploid. The genetics of the crop will, therefore, be based not only on the genetics of its diploid progenitors but also on the genetic interactions between the constituent genomes, in both pure line and hybrid breeding (Washburn and Birchler, 2013; Santantonio et al., 2019). Genetic interactions can be between alleles at a single locus or epistatically between pairs of or many non-allelic loci inter- or intrachromosomally (Jiang et al., 2017; Mackay et al., 2021). Such interactions could be within the nucleus affecting transcriptional and posttranscriptional regulatory steps but could also be between pathways and networks underpinning interacting traits (Schnell and Cockerham, 1992; Gimenez et al., 2021). No doubt numerous mechanisms giving rise to positive and negative attributes are frequent in nature. This perspective explores some recent findings to update ways of thinking about polyploid wheat genetics and crop improvement. Speculations are necessary but these are the substance of testable hypotheses.

### Interchromosomal Genetic Interactions Correlating With Negative Heterosis

**FIGURE 1:**
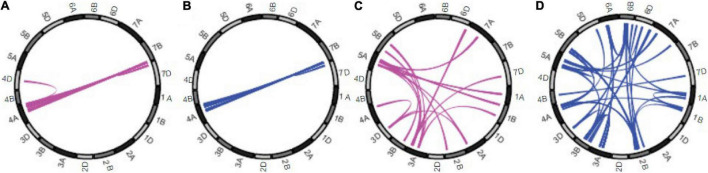
Genetic architecture of mid-parent heterosis for grain yield. Results for the 21 wheat chromosomes shown around the circumferences. Colored links in the centers of the circles represent significant digenic epistatic interactions: additive by dominance for elite hybrids **(A)** and for exotic × elite **(C)**, dominance by dominance for elite hybrids **(B)** and for exotic × elite **(D)**. Taken from Boeven et al. (2020).

European wheat breeders studying the genetic interactions underlying heterosis for yield found that the heterosis was mostly due to epistatic interactions (Jiang et al., 2017). Digenic interactions involving the A and B genomes were more frequent than those with the D genomes. In a subsequent study, European winter wheat breeders compared the heterotic interactions between progeny of elite × elite and elite × exotic crosses (Boeven et al., 2020). There was a significant difference between the two groups in the genetic architecture of the heterosis detected (Figure 1). Additive by additive epistatic interactions were the major origins of heterosis in the elite hybrids, while dominance and epistatic, including positive and especially, negative, dominance interactions were more common in the exotic × elite crosses. Mapping of the digenic epistatic interactions underlying these results showed that in the case of the exotic × elite crosses, there were many epistatic associations between different chromosomes, including non-homologous, but almost none in the elite × elite hybrids (Figure 1). Thus, negative heterosis and interchromosomal interactions were correlated.

Although few digenic epistatic QTL interactions between non-homologous chromosomes were found in crosses between the high-yielding elite lines (Boeven et al., 2020), some interactions remained between chromosomes 4A and 7B (Figure 1). More interactions were recorded in a different set of hybrids (Jiang et al., 2017) but again many involved chromosomes 4A and 7B. This is very significant because these chromosomes have undergone reciprocal translocations during hexaploid wheat evolution (Dvorak et al., 2018). Thus, it appears that chromosome segments whose origins are in the same chromosome arm retain interactions in high-yielding wheat even when the segments are translocated to a chromosome originally belonging to another diploid progenitor. That these interactions have not been selected away during intensive wheat breeding endorses the conclusion that certain chromosomal interactions in the nucleus are important.

### Chromatin Interactions in Nuclei and Gene Expression

Heterosis as defined by the geneticist has interactions at its heart. During the past decade, we have become aware of specific interactions on a massive scale between loci closely linked, between genes mapping further apart, and even on different chromosomes due to the development of new technologies that enable nearby sequences within nuclei to be identified (e.g., Peng et al., 2019; Concia et al., 2020; Jia et al., 2021; Pei et al., 2021). Such interactions have been recently assessed within and between hexaploid wheat chromosomes (Concia et al., 2020; Jia et al., 2021).

**FIGURE 2:**
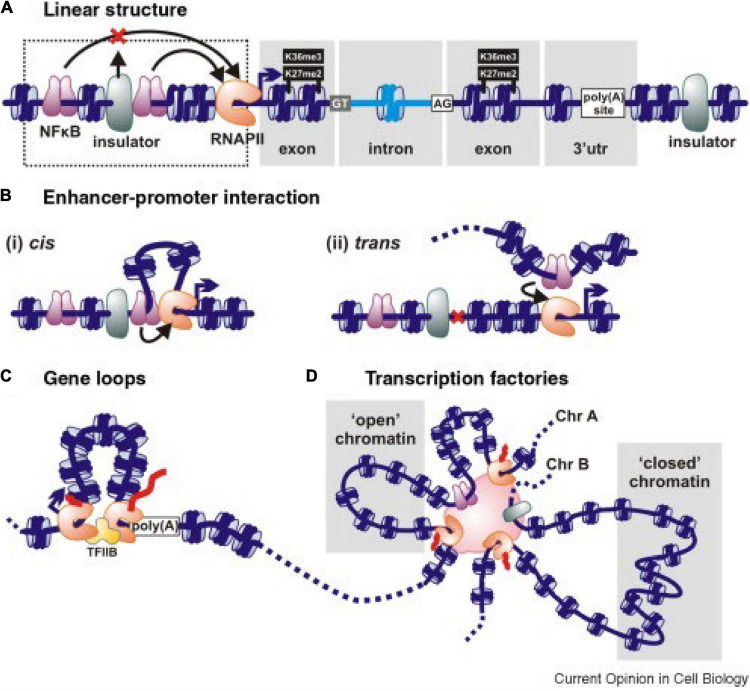
Schematic interactions associated with gene transcription. **(A)** A linear representation of genetic elements including an insulator that prevents an upstream regulatory sequence NFkB affecting downstream gene expression. Histones in nucleosomes can be methylated at specific sites, as illustrated. DNA sequences can also be methylated at cytosine residues. **(B)** Enhancer–promoter interactions deployed in cis (i) to generate a local loop or *in trans* (ii) between chromosomes stimulate transcription. Nucleosomes are in blue, and transcription factors are in purple. **(C)** Gene loop. The 5′ and 3′ ends of an active gene are juxtaposed and tied by RNA polymerase (pink-brown) and/or transcription factors (TFIIB here). **(D)** A polymorphic transcription factory showing how transcription units on the same or different chromosomes (Chr) are bound through RNA polymerases or transcription factors. “Open” chromatin is transcribed when promoters in it attach to the factory; “closed” chromatin is remote from the factory and transcriptionally silent. The enhancers of multiple genes within the same transcription factory can come under the influence of any/some of the transcription factors concentrated in the factory. Taken from Papantonis and Cook (2010).

In hexaploid wheat interphase nuclei, folded, condensed chromatin segments enriched in cytosine methylation and histone H3K27me3 are common. Some 32,000 such structures, comprising 51% of the genome, were found by Concia et al. (2020), with an average length of 225 kbp. They form “intergenic condensed spacers” separating gene-rich regions having less condensed chromatin and less DNA methylation but enriched with histones H3K9ac and H3K36me3. These gene-rich chromatin regions form loops at the boundaries of the condensed intergenic spacers (Figure 2C). Loop formation is the mechanism by which regulatory pieces of chromatin distant from a gene (Figure 2A) are brought into close proximity to promoters and other pieces of chromatin to initiate and regulate transcription (Figures 2Bi,ii; Schoenfelder et al., 2010; Cook and Marenduzzo, 2018; Peng et al., 2019). The frequency of intrachromosomal loop formation is often higher than between chromosomes. However, large numbers of interchromosomal associations also occur, preferentially based on similar sequences that lead to associations within subgenomes rather than between subgenomes in this allopolyploid (Concia et al., 2020; Jia et al., 2021). Gene–gene loop associations are also common. Overall, 29% of all wheat genes were found to be associated with one or more gene–gene loops, and genes in gene–gene loops displayed similar expression levels, i.e., were co-regulated (Concia et al., 2020). The extent of co-regulation was stronger for gene–gene loops associated with RNA polymerase ll; 50% of genes associated with RNA polymerase ll had 4 or more partners, and 11% of genes associated with RNA polymerase ll had 10 or more partners. In some instances, there were up to 20 genes associated together in addition to RNA polymerase ll.

Because loops and loop complexes are associated with RNA Polymerase ll, it is concluded that they form “transcription factories” (TFs) (Figure 2D; Schoenfelder et al., 2009; Sutherland and Bickmore, 2009; Concia et al., 2020). These factories are assumed to include many other molecules, such as coactivators, chromatin remodelers, transcription factors, histone modification enzymes, RNPs, RNAs, non-coding RNAs, helicases, splicing, and processing factors associated with transcription and RNA processing (Tsai et al., 2020; Bertero, 2021). It has been proposed that genes and nearby sequences become clustered into a TF by their common affinity for and accessibility to specific transcription factors, RNA polymerases, and other cofactors (Figure 2D). There is also discussion that such factories enable much higher concentrations of RNA polymerases, transcription factors, and other cofactors to be achieved, thereby increasing the efficiency of transcription (Sutherland and Bickmore, 2009; Cook and Marenduzzo, 2018; Tsai et al., 2020). Incorporation of genes into transcription factories is not random, but regulated. It can be assumed that the chromatin interactions in TFs are specific and subject to natural and artificial selection in breeding programs.

The coordinated expression of genes in associated loops (in the same transcription factory) implies that *in trans* interactions occur within TFs involving enhancers, promoters, RNA polymerase, regulatory proteins, coding and non-coding RNA transcripts, R loop formation, and RNA processing systems (Bertero, 2021) to mutually influence the outputs of expression of the genes in each factory (Schoenfelder et al., 2009; Cook and Marenduzzo, 2018; Zhang et al., 2019). Perturbations of these interactions by incorporation of a genetic variant into the TF, possibly created by variation in transposable elements with binding affinity for transcription factors (Zhang et al., 2021) or one with a different 3D loop structure, could result in each gene being expressed to the same, greater or a lower extent because of the interactions (Cook and Marenduzzo, 2018). Such perturbations may be a major source of phenotypic variation defined by quantitative genetics as non-additive, dominant, overdominant, or epistatic, positively or negatively; and the interfering DNAs would map as QTLs or eQTLs (Cook and Marenduzzo, 2018; Delaneau et al., 2019; Peng et al., 2019; Zhao et al., 2019; Tsai et al., 2020). Several examples of correlations between physical interactions and subgenome dominance, colocalization of paralogs and homologs, and biased gene retention after polyploidization in plants are reviewed in Huang et al. (2020). Regulation within a transcription factory, therefore, appears to form a “middle layer” of regulation between (a) the molecular control of expression of individual genes and (b) the complex integration of gene networks, physiology, and biochemistry, determining phenotypic traits at the cell and tissue/organ levels (Tsai et al., 2020). The biology of TFs is likely to have profound importance for understanding the genetic basis of traits and plant breeding. They may also be sources of the large number of QTLs that contribute very small proportions of the genetic variation underpinning a trait, that map all across the genome, and that were highlighted by Boyle et al. (2017) in their “omnigenic” model for trait determination in the human genome.

### Epigenetics and Transcription Factory Formation

The efficiency with which a gene loop is formed in a transcription factory is determined by its epigenetic state, among other factors, which in turn is determined by its DNA sequence and the transacting factors which specify the histone methylation/acetylation and DNA methylation status of the local chromatin (Peng et al., 2019; Lv et al., 2021; Wang et al., 2021). Genetic variation that affects epigenetic features in the localities of genes, including variation due to transposable elements carrying binding sites for transcription factors (Zhang et al., 2021), is therefore likely to influence loop formation and transcription factory structures (Peng et al., 2019).

A genome-wide atlas of the epigenetic states of genes/promoters and enhancers has been created for wheat by pairing the epigenetic states of the chromatin using the histone marks H3K4me3 and H3k9ac/H3K27ac and the repressive mark H3K27me3, which all vary with gene activity (Wang et al., 2021). For around 80,000 genes, 224,000 regulatory elements were linked to specific promoters within 500 kb, and these linkages correlated highly with the physical loop linkages published by Concia et al. (2020); 67% of the promoters/genes were linked with more than one regulatory element, with 50% having 2–10 elements, 9% having 11 to twenty elements, and 7% having more than twenty elements, indicating the combinatorial regulation of gene activity by multiple *cis*-linked regulatory elements. These results have significant implications for haplotype breeding approaches (Brinton et al., 2020). The epigenetic states of each of the elements were tied to developmental and environmental conditions and some to different organs, e.g., spikes. They also revealed the genes in the subgenomes of wheat that were differentially expressed in contrast to alleles that were expressed similarly in all three subgenomes (Wang et al., 2021).

Changes in the epigenetic status and relative activities of genes, when brought together in new wheat hybrids, have been recognized for a long time in the case of the ribosomal RNA genes, which are found to be associated with nucleoli (Sardana et al., 1993). Nucleoli are the most well-known and most-studied transcription factories. My group’s studies into these sets of genes and transcription factories in wheat, as well as those of others, demonstrated that when different nucleolar organizers (NORs) comprising arrays of rRNA genes are brought together *via* a genetic cross, certain rDNA loci become active/dominant or semi-dominant over others which become relatively silent. Dominance is associated with open chromatin and lower cytosine methylation, while repression is associated with condensed chromatin and higher methylation. The degree of dominance appears to correlate with the number of promoters/enhancers in the regions upstream of the major rRNA gene promoter in the locus (Sardana et al., 1993) and complex transcription patterns of the upstream enhancer regions by RNA polymerase 1 and/or RNA polymerase ll (Vincentz and Flavell, 1989; Earley et al., 2010; Abraham et al., 2020). The differential epigenetic state of the different NORs is likely mediated by siRNAs, generated from the more active transcribed NOR in a TF, acting *in trans* to silence or reduce the activity of other NORs (Preuss et al., 2008; Earley et al., 2010).

The epigenetic profiles of the different rDNA loci (NORs) are established in the egg and early zygote divisions when the parental genomes are first brought together and are inherited through subsequent somatic cell divisions until another meiosis or different regulatory situation is imposed. Thus, the epigenetic chromatin state of an allele reflects the outcome of its activity relative to other loci when they are first active together in the F1 individual plant. Resulting siRNAs may change the chromatin structures of alleles differentially and, consequently, an allele’s ability to be incorporated into and expression in a TF until other developmental regulatory factors emerge to alter its epigenetic state.

### Implications for Plant Breeding

The conclusion that selections in European wheat in recent decades have purged epistatic sources of negative dominance effects (Jiang et al., 2017; Boeven et al., 2020) prompts the questions (i) whether yield gains over recent decades have predominantly been due to this elimination and, if so, (ii) whether yield gains have now slowed due to elimination of most of the readily deleted, deleterious variation and the remaining negative heterotic effects are too difficult to remove because of linkage to very favorable alleles or some other reason. Additional explanations for the slow rates of gain are, of course, the failure to incorporate new variation into the elite breeding pools, the genetic complexity of yield, the climate becoming more variable, or the application of different agricultural policies (Calderini and Slafer, 1999; Brisson et al., 2010; Lin and Huybers, 2012).

Genetic variation on which plant breeding is based will be predominantly found in genes and their regulatory elements, including the regulatory elements in transposable elements (Zhang et al., 2021). The availability of an atlas of mapped wheat genes and the regulatory elements with which they are epigenetically paired (Wang et al., 2021) offers the opportunity to define QTLs in terms of genes and paired regulatory elements. The knowledge that single genes are frequently associated with multiple regulatory elements (Wang et al., 2021) predicts multiple potential QTLs in each subgenome of wheat for a trait determined by that single gene. Furthermore, the finding that a high proportion of (but not all) active genes are in physical associations, whether genetically linked or not, and in consequence become co-regulated with other genes in transcription factories (Concia et al., 2020) further increases the numbers of variables and hence potential QTLs affecting gene expression. All these findings help explain why the phenotypic effects of single genes vary with genetic background (including unlinked genes) as is commonly found.

Knowledge of the physical interactions of genes and regulatory elements, including those more distant in transposable elements (Zhang et al., 2021) in TFs, makes it tempting to explain at least some of the interactions detected genetically, heterosis for example, as the result of such physical interactions. Highlighted in this perspective on wheat are the higher digenic mapping interactions between A and B genomes than with D genomes (Jiang et al., 2017) and the higher incidence of physical interactions between A and B genomes compared with those involving the D genome (Concia et al., 2020; Jia et al., 2021). This is likely to be due to the opportunities for the establishment and selection of functional links between the A and B genomes during their long association in tetraploid wheat relative to the much shorter time in which the D genome has been present in hexaploid wheat (Concia et al., 2020; Jia et al., 2021). Genes become associated with TFs based on their epigenetic profiles that determine their availability for interactions. Thus, the control systems that determine the methylation of DNA and histones are key, and these can clearly generate new outcomes, including the participation of genes in TFs, upon the formation of new genotypes in crosses. The outcomes of these epigenetic modulations are likely to be a frequent basis of dominance, semi-dominance, and recessive classifications (Schoenfelder et al., 2009; Cook and Marenduzzo, 2018) and can clearly vary between subgenomes in polyploids, genetic backgrounds, and through development (Wang et al., 2021).

Because genes in a given TF are co-regulated, uncovering genes within a transcription factory may help establish hypotheses that link genes, transcription factors, factories, and traits and extend the data on gene networks in KnetMiner.^1^ If genes within a factory are co-selected and coordinated to contribute to the same trait, then studying the variation between genotypes for a given TF would provide a rapid way of defining targets for trait improvement, either by genetic intervention using CRISPR/Cas technologies or by selection using defined DNA markers. These are testable hypotheses. Comparisons between diverse individuals of genes and their regulatory elements in functionally equivalent TFs and the haplotypic segments associated with them should enable alleles that poison or enhance factories to be identified and also become targets for elimination, exchange, modification, or selection. In a polyploid like wheat, any such identified genes could perhaps be deleted or inactivated without major detriment using CRISPR/Cas-based technologies, thereby deleting sources of negative heterosis in otherwise excellent parents of F1 hybrids. This is a testable approach. Where detrimental sequences are too numerous to be deleted, then it may be possible to edit the favorable sequences into other lines to create a genetic gain. The hypothesis that genes not in factories are much less likely to be involved in determining heterosis can also be tested. Genes belonging to more than one factory may be particularly interesting and important to characterize.

Interacting gene-containing loops in TFs are most often from the same chromosome, which implies that the loops are genetically linked. Recombination between them will therefore be infrequent in breeding programs. This limits the opportunities for exploiting homologous recombination to modify the content of TFs in pure line breeding. Hybrids would be better for creating new combinations of alleles/genes in TFs, especially where intrachromosomal interactions are the most frequent. Because TFs are likely to include unlinked genes, including genes on homologous and non-homologous chromosomes, variant TFs created in breeding programs are most likely to result from combining variant genes and their regulatory elements from different chromosomes. Given the above logic, it is not surprising that much/most of the variations underpinning heterosis in wheat are epistatic (Jiang et al., 2017; Santantonio et al., 2019; Boeven et al., 2020) and arise from unlinked genes, including non-homologous genes. Furthermore, the greater the proportion of unlinked genes within a TF, the greater the probability that the TF will be a source of phenotypic variation in hybrids and fixable in pure line breeding programs.

While breeders are often reluctant to lower the performance of elite breeding pools by making wide crosses, there are many examples of yield gains being achieved by the incorporation into domesticated wheat varieties of a trait-enhancing chromosomal segment or segments from wild relatives (Ceoloni et al., 2017). Yet, it is unlikely that there are no negative genetic elements in such selections, even when the segments have been reduced in size by recombination and selection. Therefore, there is a strong case for deploying where possible a deletion strategy on such segments *via* CRISPR/Cas editing to delete or correct the remaining genes/regulatory elements that epistatically inhibit yield, thereby increasing the segments’ value even more. Deletion breeding based on precision-targeted editing needs to be explored. It also may be the means by which new variation can be continuously brought into elite breeding pools without negative yield losses, thereby boosting continuous yield gains over long periods. In addition, the deployment of new enhancers to genes that are incorporated into TFs using CRISPR/Cas or transgenic technologies may also provide new opportunities to enhance the expression of the multiple genes within the TF, thereby enhancing the traits in a more efficient way than by changing one gene at a time.

## Data Availability Statement

The original contributions presented in the study are included in the article/supplementary material, further inquiries can be directed to the corresponding author/s.

## Author Contributions

The author confirms being the sole contributor of this work and has approved it for publication.

## Conflict of Interest

The author declares that the research was conducted in the absence of any commercial or financial relationships that could be construed as a potential conflict of interest.

## Publisher’s Note

All claims expressed in this article are solely those of the authors and do not necessarily represent those of their affiliated organizations, or those of the publisher, the editors and the reviewers. Any product that may be evaluated in this article, or claim that may be made by its manufacturer, is not guaranteed or endorsed by the publisher.

## Footnotes

1
https://knetminer.org/Triticum_aestivum/
